# Retraction: Adverse Pregnancy Outcomes Following Exposure to Biologics in Women With Crohn's Disease: A Systematic Review and Meta-Analysis

**DOI:** 10.3389/fmed.2022.915112

**Published:** 2022-04-21

**Authors:** Frontiers Editorial Office

The cited article had been retracted.

Following publication, concerns were raised regarding the accuracy and validity of the original data presented in this article. The authors subsequently contacted the Editorial Office to request the retraction of the article, stating that technical errors in the inclusion criteria for the initial literature screening and data extraction do not sufficiently support the reported conclusions. This was confirmed in our investigation, conducted in accordance with our policies.

This retraction was approved by the Chief Editors of Frontiers in Medicine and the Chief Executive Editor of Frontiers. The authors agree to this retraction.

